# 2-Chloro­quinoline-3-carboxylic acid

**DOI:** 10.1107/S1600536810006501

**Published:** 2010-02-27

**Authors:** Souheila Ladraa, Abdelmalek Bouraiou, Sofiane Bouacida, Thierry Roisnel, Ali Belfaitah

**Affiliations:** a Laboratoire des Produits Naturels d’Origine Végétale et de Synthèse Organique, PHYSYNOR, Université Mentouri-Constantine, 25000 Constantine, Algeria; b Unité de Recherche de Chimie de l’Environnement et Moléculaire Structurale, CHEMS, Université Mentouri-Constantine, 25000 Algeria; c Centre de difractométrie X, UMR 6226 CNRS Unité Sciences Chimiques de Rennes, Université de Rennes I, 263 Avenue du Général Leclerc, 35042 Rennes, France

## Abstract

The crystal structure of the title compound, C_10_H_6_ClNO_2_, can be described by two types of crossed layers which are parallel to (110) and (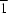
10). The crystal packing is stabilized by inter­molecular C—H⋯O and O—H⋯N hydrogen bonds, resulting in the formation of a two-dimensional network and reinforcing the cohesion of the structure.

## Related literature

For our previous work on the preparation of α-amino­nitriles, see: Ladraa *et al.* (2009 ▶); Belfaitah *et al.* (2006 ▶). For the removal of chiral auxiliaries using ceric ammonium nitrate, see: Bhanu Prasad *et al.* (2004 ▶).
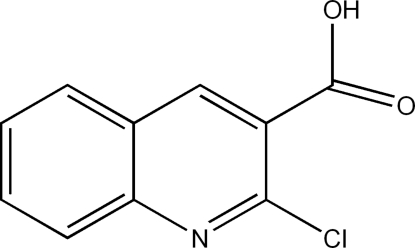


## Experimental

### 

#### Crystal data

C_10_H_6_ClNO_2_
*M* *_r_* = 207.61
Orthorhombic, 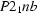

*a* = 5.8193 (2) Å
*b* = 8.0689 (3) Å
*c* = 18.1780 (5) Å
*V* = 853.55 (5) Å^3^
*Z* = 4
Mo *K*α radiation
μ = 0.41 mm^−1^
*T* = 120 K
0.19 × 0.12 × 0.08 mm

#### Data collection

Nonius KappaCCD diffractometer
Absorption correction: multi-scan (*SADABS*; Sheldrick, 2002 ▶) *T*_min_ = 0.915, *T*_max_ = 0.967
13714 measured reflections
1938 independent reflections
1746 reflections with *I* > 2σ(*I*)
*R*_int_ = 0.046

#### Refinement

*R*[*F* ^2^ > 2σ(*F* ^2^)] = 0.038
*wR*(*F* ^2^) = 0.094
*S* = 1.14
1938 reflections
129 parameters
1 restraint
H-atom parameters constrained
Δρ_max_ = 0.32 e Å^−3^
Δρ_min_ = −0.35 e Å^−3^
Absolute structure: Flack (1983 ▶), 862 Friedel pairs
Flack parameter: 0.28 (9)

### 

Data collection: *COLLECT* (Nonius, 1998 ▶); cell refinement: *SCALEPACK* (Otwinowski & Minor, 1997 ▶); data reduction: *DENZO* (Otwinowski & Minor 1997 ▶) and *SCALEPACK*; program(s) used to solve structure: *SIR2002* (Burla *et al.*, 2003 ▶); program(s) used to refine structure: *SHELXL97* (Sheldrick, 2008 ▶); molecular graphics: *ORTEP-3 for Windows* (Farrugia, 1997 ▶) and *DIAMOND* (Brandenburg & Berndt, 2001 ▶); software used to prepare material for publication: *WinGX* (Farrugia, 1999 ▶).

## Supplementary Material

Crystal structure: contains datablocks global, I. DOI: 10.1107/S1600536810006501/bq2197sup1.cif

Structure factors: contains datablocks I. DOI: 10.1107/S1600536810006501/bq2197Isup2.hkl

Additional supplementary materials:  crystallographic information; 3D view; checkCIF report

## Figures and Tables

**Table 1:** Hydrogen-bond geometry (Å, °)

*D*—H⋯*A*	*D*—H	H⋯*A*	*D*⋯*A*	*D*—H⋯*A*
O2—H2⋯N1^i^	0.84	1.95	2.768 (3)	164
C8—H8⋯O1^ii^	0.95	2.37	3.290 (4)	163

Symmetry codes: (i) 
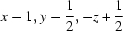
; (ii) 
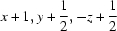
.

**Fig. 1.**
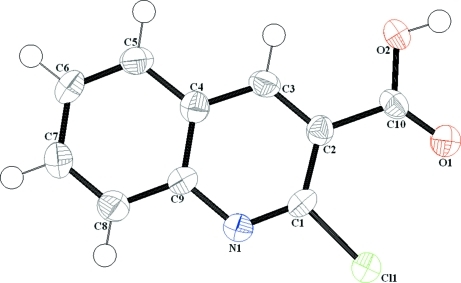
(Farrugia, 1997) the structure of the title compound with the atomic labeling scheme. Displacements are drawn at the 50% probability level.

**Fig. 2.**
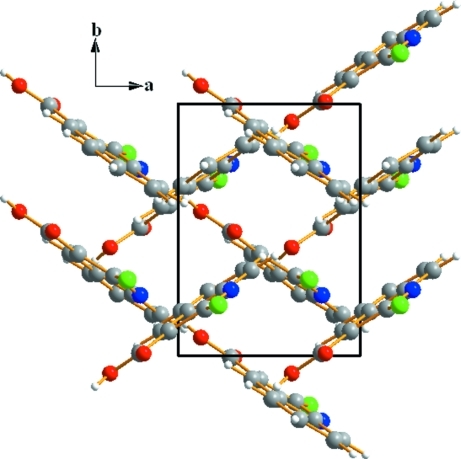
(Brandenburg & Berndt, 2001) A diagram of the layered crystal packing of (I) viewed down the c axis.

**Fig. 3.**
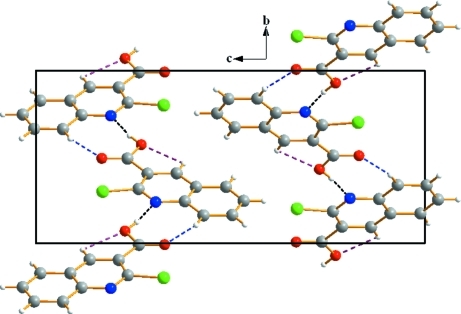
(Brandenburg & Berndt, 2001) Unit cell of (I) showing hydrogen bond [O—H···N and C—H···O] as dashed line.

## supplementary crystallographic information

### Comment

In a continuation of our previous work related to the preparation of α-aminonitrile (Ladraa *et al.*, 2009; Belfaitah *et al.*, 2006) and in order to prepare chiral N-deprotected α-aminonitrile, we have explored the oxidative debenzylation of N-protected α-aminonitrile. The removal of the chiral auxiliaries has already been investigated using ceric ammonium nitrate (CAN) (Bhanu Prasad *et al.*, 2004). Surprisingly, our attempts to remove the chiral auxiliary using CAN were failed to undergo the desired adduct and led to the 2-chloroquinoline-3-carboxylic acid (I). This unexpected cleavage of 2-[(*S*)-2-chloro-3-quinolyl]-2-[(*R*)-1-(4-methoxyphenyl) ethylamino]acetonitrile may result from the decomposition of the α-aminonitrile into cyanide and imine which in turn undergo hydrolysis/oxidative sequence. In this paper, we report the structure determination of compound (I), resulting from unwanted decomposition of chiral N-protected α-aminonitrile.

The molecular geometry and the atom-numbering scheme of (I) are shown in Fig. 1. The two rings of quinolyl moiety are fused in an axial fashion and form a dihedral angle of 0.42 (9)°. The crystal packing can be described by two types of crossed layers which quinolyl ring is parallel to (110) and (-110) planes respectively (Fig. 2). The crystal packing is stabilized by inter and intramolecular hydrogen bonds (O—H···N and C—H···O) linked molecules in the same layer, resulting in the formation of a two dimensional network and reinforcing a cohesion of structure. Hydrogen-bonding parameters are listed in table 1.

### Experimental

A solution of 327 mg (3 eq., 0.59 mmol.) of ceric ammonium nitrate (CAN) in 1 ml of water was added to precooled stirred solution of 2-[(*S*)-2-chloro-3-quinolyl]-2-[(*R*)-1-(4-methoxyphenyl) ethylamino] acetonitrile (70 mg, 0.19 mmol) in 9 ml of CH_3_CN. After completion of the reaction (checked by TLC), the reaction mixture was poured into cold water and the residue obtained was filtered off. Crystals of (I) suitable for X-ray analysis were obtained by slow evaporation of the filtrate.

### Refinement

All non-H atoms were refined with anisotropic atomic displacement parameters. All H atoms were localized on Fourier maps but introduced in calculated positions and treated as riding on their parent C and O atoms. (with C—H = 0.95Å - O—H =0.84Å and U_iso_(H) =1.2 or 1.5(carrier atom)).

### Crystal data

**Table:**

C_10_H_6_ClNO_2_	*F*(000) = 424
*M**_r_* = 207.61	*D*_x_ = 1.616 Mg m^−^^3^
Orthorhombic, *P*2_1_*n**b*	Mo *K*α radiation, λ = 0.71073 Å
Hall symbol: P -2bc 2a	Cell parameters from 21289 reflections
*a* = 5.8193 (2) Å	θ = 2.9–27.5°
*b* = 8.0689 (3) Å	µ = 0.41 mm^−^^1^
*c* = 18.1780 (5) Å	*T* = 120 K
*V* = 853.55 (5) Å^3^	Prism, yellow
*Z* = 4	0.19 × 0.12 × 0.08 mm

### Data collection

**Table:**

Nonius KappaCCD diffractometer	1938 independent reflections
Radiation source: Enraf–Nonius FR590	1746 reflections with *I* > 2σ(*I*)
graphite	*R*_int_ = 0.046
Detector resolution: 9 pixels mm^-1^	θ_max_ = 27.5°, θ_min_ = 3.4°
CCD rotation images, thin slices scans	*h* = −7→7
Absorption correction: multi-scan (*SADABS*; Sheldrick, 2002)	*k* = −10→10
*T*_min_ = 0.915, *T*_max_ = 0.967	*l* = −23→23
13714 measured reflections	

### Refinement

**Table:**

Refinement on *F*^2^	Secondary atom site location: difference Fourier map
Least-squares matrix: full	Hydrogen site location: inferred from neighbouring sites
*R*[*F*^2^ > 2σ(*F*^2^)] = 0.038	H-atom parameters constrained
*wR*(*F*^2^) = 0.094	*w* = 1/[σ^2^(*F*_o_^2^) + (0.0388*P*)^2^ + 0.7398*P*] where *P* = (*F*_o_^2^ + 2*F*_c_^2^)/3
*S* = 1.14	(Δ/σ)_max_ = 0.001
1938 reflections	Δρ_max_ = 0.32 e Å^−^^3^
129 parameters	Δρ_min_ = −0.35 e Å^−^^3^
1 restraint	Absolute structure: Flack (1983), 862 Friedel pairs
Primary atom site location: structure-invariant direct methods	Flack parameter: 0.28 (9)

### Special details

**Table:**

Geometry. All esds (except the esd in the dihedral angle between two l.s. planes) are estimated using the full covariance matrix. The cell esds are taken into account individually in the estimation of esds in distances, angles and torsion angles; correlations between esds in cell parameters are only used when they are defined by crystal symmetry. An approximate (isotropic) treatment of cell esds is used for estimating esds involving l.s. planes.
Refinement. Refinement of *F*^2^ against ALL reflections. The weighted *R*-factor wR and goodness of fit *S* are based on *F*^2^, conventional *R*-factors *R* are based on *F*, with *F* set to zero for negative *F*^2^. The threshold expression of *F*^2^ > σ(*F*^2^) is used only for calculating *R*-factors(gt) etc. and is not relevant to the choice of reflections for refinement. *R*-factors based on *F*^2^ are statistically about twice as large as those based on *F*, and *R*- factors based on ALL data will be even larger.

### Fractional atomic coordinates and isotropic or equivalent isotropic displacement parameters (Å^2^)

**Table:**

	*x*	*y*	*z*	*U*_iso_*/*U*_eq_	
C1	0.1466 (5)	0.1851 (3)	0.23620 (14)	0.0167 (5)	
C2	−0.0521 (4)	0.0966 (3)	0.21287 (14)	0.0161 (5)	
C3	−0.0939 (5)	0.0943 (3)	0.13814 (14)	0.0182 (5)	
H3	−0.2255	0.0377	0.1201	0.022*	
C4	0.0535 (5)	0.1735 (3)	0.08831 (15)	0.0175 (5)	
C5	0.0162 (5)	0.1732 (3)	0.01110 (15)	0.0204 (6)	
H5	−0.113	0.1173	−0.009	0.024*	
C6	0.1668 (5)	0.2538 (3)	−0.03428 (14)	0.0196 (6)	
H6	0.1416	0.254	−0.0859	0.024*	
C7	0.3592 (5)	0.3363 (3)	−0.00485 (15)	0.0217 (6)	
H7	0.4622	0.3918	−0.0371	0.026*	
C8	0.4009 (5)	0.3382 (3)	0.06923 (15)	0.0200 (6)	
H8	0.5315	0.3945	0.0882	0.024*	
C9	0.2487 (5)	0.2563 (3)	0.11715 (13)	0.0164 (5)	
C10	−0.2112 (4)	0.0090 (3)	0.26469 (13)	0.0168 (5)	
N1	0.2901 (4)	0.2608 (3)	0.19237 (12)	0.0173 (5)	
O1	−0.1908 (4)	0.0077 (3)	0.33080 (10)	0.0268 (5)	
O2	−0.3754 (4)	−0.0713 (3)	0.22913 (11)	0.0256 (5)	
H2	−0.4571	−0.1233	0.2594	0.038*	
Cl1	0.22006 (13)	0.19998 (8)	0.32850 (3)	0.02526 (17)	

### Atomic displacement parameters (Å^2^)

**Table:**

	*U*^11^	*U*^22^	*U*^33^	*U*^12^	*U*^13^	*U*^23^
C1	0.0190 (13)	0.0187 (13)	0.0124 (11)	0.0014 (10)	−0.0030 (9)	−0.0007 (10)
C2	0.0152 (13)	0.0183 (12)	0.0148 (13)	0.0012 (10)	0.0014 (9)	0.0008 (10)
C3	0.0137 (13)	0.0211 (13)	0.0198 (13)	−0.0030 (10)	0.0003 (11)	−0.0030 (10)
C4	0.0168 (13)	0.0180 (12)	0.0176 (12)	0.0023 (10)	0.0013 (10)	−0.0001 (10)
C5	0.0183 (14)	0.0233 (13)	0.0195 (13)	−0.0025 (11)	−0.0025 (11)	−0.0032 (11)
C6	0.0235 (17)	0.0227 (13)	0.0127 (12)	0.0034 (10)	−0.0010 (10)	−0.0001 (9)
C7	0.0221 (15)	0.0239 (14)	0.0191 (13)	−0.0008 (11)	0.0041 (11)	0.0029 (11)
C8	0.0174 (14)	0.0220 (13)	0.0208 (13)	0.0003 (11)	−0.0002 (10)	−0.0002 (11)
C9	0.0170 (14)	0.0169 (11)	0.0151 (11)	0.0006 (11)	−0.0019 (11)	−0.0008 (8)
C10	0.0142 (13)	0.0168 (12)	0.0195 (13)	−0.0022 (10)	0.0035 (10)	−0.0008 (10)
N1	0.0171 (11)	0.0176 (10)	0.0173 (10)	−0.0023 (8)	−0.0008 (9)	−0.0002 (9)
O1	0.0259 (10)	0.0380 (12)	0.0165 (9)	−0.0116 (9)	0.0015 (8)	−0.0001 (8)
O2	0.0242 (11)	0.0347 (11)	0.0180 (10)	−0.0156 (9)	0.0012 (8)	0.0041 (9)
Cl1	0.0242 (3)	0.0352 (3)	0.0164 (3)	−0.0106 (3)	−0.0016 (3)	0.0029 (2)

### Geometric parameters (Å, °)

**Table:**

C1—N1	1.306 (3)	C6—C7	1.408 (4)
C1—C2	1.424 (4)	C6—H6	0.95
C1—Cl1	1.736 (3)	C7—C8	1.368 (4)
C2—C3	1.380 (4)	C7—H7	0.95
C2—C10	1.498 (3)	C8—C9	1.407 (4)
C3—C4	1.402 (4)	C8—H8	0.95
C3—H3	0.95	C9—N1	1.389 (3)
C4—C9	1.418 (4)	C10—O1	1.208 (3)
C4—C5	1.420 (4)	C10—O2	1.323 (3)
C5—C6	1.368 (4)	O2—H2	0.84
C5—H5	0.95		
			
N1—C1—C2	124.9 (2)	C5—C6—H6	119.8
N1—C1—Cl1	113.6 (2)	C7—C6—H6	119.8
C2—C1—Cl1	121.5 (2)	C8—C7—C6	121.3 (3)
C3—C2—C1	116.3 (2)	C8—C7—H7	119.3
C3—C2—C10	120.2 (2)	C6—C7—H7	119.3
C1—C2—C10	123.5 (2)	C7—C8—C9	119.5 (3)
C2—C3—C4	121.5 (3)	C7—C8—H8	120.2
C2—C3—H3	119.3	C9—C8—H8	120.2
C4—C3—H3	119.3	N1—C9—C8	119.2 (2)
C3—C4—C9	117.8 (2)	N1—C9—C4	121.0 (2)
C3—C4—C5	123.0 (3)	C8—C9—C4	119.8 (2)
C9—C4—C5	119.2 (2)	O1—C10—O2	123.6 (2)
C6—C5—C4	119.8 (3)	O1—C10—C2	124.7 (2)
C6—C5—H5	120.1	O2—C10—C2	111.7 (2)
C4—C5—H5	120.1	C1—N1—C9	118.5 (2)
C5—C6—C7	120.3 (2)	C10—O2—H2	109.5
			
N1—C1—C2—C3	−0.8 (4)	C7—C8—C9—C4	−0.3 (4)
Cl1—C1—C2—C3	179.6 (2)	C3—C4—C9—N1	−1.0 (4)
N1—C1—C2—C10	179.0 (2)	C5—C4—C9—N1	179.4 (2)
Cl1—C1—C2—C10	−0.6 (4)	C3—C4—C9—C8	−179.7 (2)
C1—C2—C3—C4	0.7 (4)	C5—C4—C9—C8	0.7 (4)
C10—C2—C3—C4	−179.2 (2)	C3—C2—C10—O1	−178.2 (3)
C2—C3—C4—C9	0.2 (4)	C1—C2—C10—O1	1.9 (4)
C2—C3—C4—C5	179.8 (3)	C3—C2—C10—O2	3.3 (4)
C3—C4—C5—C6	179.7 (3)	C1—C2—C10—O2	−176.6 (2)
C9—C4—C5—C6	−0.6 (4)	C2—C1—N1—C9	0.1 (4)
C4—C5—C6—C7	0.3 (4)	Cl1—C1—N1—C9	179.68 (18)
C5—C6—C7—C8	0.1 (4)	C8—C9—N1—C1	179.6 (2)
C6—C7—C8—C9	0.0 (4)	C4—C9—N1—C1	0.9 (4)
C7—C8—C9—N1	−179.0 (3)		

### Hydrogen-bond geometry (Å, °)

**Table:**

*D*—H···*A*	*D*—H	H···*A*	*D*···*A*	*D*—H···*A*
O2—H2···N1^i^	0.84	1.95	2.768 (3)	164
C3—H3···O2	0.95	2.34	2.685 (4)	101
C8—H8···O1^ii^	0.95	2.37	3.290 (4)	163

Symmetry codes: (i) *x*−1, *y*−1/2, −*z*+1/2; (ii) *x*+1, *y*+1/2, −*z*+1/2.
